# Demography in Swiss paediatric uveitis: a retrospective cohort study

**DOI:** 10.1186/s12348-024-00448-4

**Published:** 2024-12-18

**Authors:** Jeanne Martine Gunzinger, Alice Kitay, Fabio Meier, Christian Böni, Seraina Palmer Sarott, Brigitte Simonsz-Tóth, Christina Gerth-Kahlert

**Affiliations:** 1 Department of Ophthalmology, University Hospital Zurich, University of Zurich, Frauenklinikstrasse 24, Zurich, 8091 Switzerland; 2 Augenzentrum Witikon, Zurich, Switzerland; 3 Augenarztpraxis Bremgarten, Bremgarten, Switzerland; 4 Department of Paediatric Rheumatology, University Children’s Hospital Zurich, Zurich, Switzerland

**Keywords:** Uveitis, Paediatric, Children, Infectious, Non-infectious, NIPU, Demographics, Switzerland

## Abstract

**Introduction:**

Paediatric uveitis is a rare disease. It can affect any segment and have various etiologies, including infectious, autoimmune, and masquerade diseases. The pupose of this study is to analyse and present the demographic data in paediatric uveitis in a Swiss cohort. Knowledge of local demography may guide targeted work up and treatment.

**Methods:**

Single center retrospective study from January 2012 to June 2022. Patients under 18 years of age with uveitis were eligible for inclusion. Demographics (age at first presentation, sex), ocular signs (affected eye segment, laterality, visual acuity; VA, Snellen, decimal, clinical course), and systemic finding were analysed. Frequencies and descriptive statistics were computed, non-parametric tests and odds ratio were applied for sample comparisons. Local ethics committee approved this study.

**Results:**

Data from 93 of 133 identified patients were available. 51% were female, mean age at first presentation was 12 years, 60% had bilateral disease. 68% were of non-infectious etiology. Most common identified etiology was toxoplasmosis (20%), followed by JIA (8%) and herpetic (8%). No associated infectious cause or systemic disease was found in 44% of the cases. Most presented with anterior uveitis (50%), followed by posterior (28%), intermediate (20%), and panuveitis (2%). 80% of anterior uveitis were non-infectious; 81% of posterior uveitis were infectious. Bilateral disease was strongly associated with non-infectious uveitis (93%), whereas unilateral disease was more likely to be of an infectious cause (70%); odds ratio = 31. Mean VA of affected eyes at first presentation was 0.79. VA was significantly worse in cases with infectious uveitis compared to non-infectious uveitis (*p* = 0.007). Nearly a third of affected eyes showed at least one complication. This did not differ between in non-infectious and infectious uveitis cases.

**Conclusion:**

Bilateral disease is strongly suggestive of non-infectious uveitis. Unilateral and posterior disease is suggestive of an infectious cause, with toxoplasmosis being the most often diagnosed cause of uveitis in this cohort. Knowledge of demography is important for specialists to target workup and introduce treatment.

## Introduction

Paediatric uveitis is a rare disease with an estimated incidence between 4.9 and 14 per 100,000 per year, but responsible for 5–25% of blindness in children and adolescents [1, 2]. In can affect any eye segment and is usually classified according to the standardization of uveitis nomenclature (SUN) in either anterior, intermediate, posterior, or panuveitis [3]. It is further divided into infectious or non-infectious uveitis. Infectious causes are far more common in children compared to adults and account for 20–35% of all paediatric uveitis cases [2]. Systemic diseases commonly associated with paediatric uveitis differ to some extent from adult uveitis [4]. In western countries, toxoplasmosis is the most common infectious cause whereas juvenile idiopathic arthritis (JIA) is the most common associated disease in non-infectious uveitis [1, 5]. Further frequently identified infectious causes are herpetic, toxocariasis, borrelia associated disease or cat-scratch disease. Other associated autoimmune diseases besides JIA are inflammatory bowel disease, psoriatic arthritis, ankylosing spondylitis, sarcoidosis, Blau syndrome, Kawasaki disease, or tubulointerstitial nephritis uveitis (TINU) syndrome [1, 6–8]. Often, no cause or associated systemic disease can be identified, varying between 34 − 59% in review from Cunningham in 2000 [1]. Treatment is aimed to control the ocular inflammation as well as targeting infectious cause or underlying autoimmune disease. It can be challenging to differentiate between infectious or non-infectious uveitis at initial presentation and during disease course as well as deciding on further systemic work up. Knowledge of the area and country specific demography may guide early decisions in work up and treatment. This paper discusses the baseline demographics of children presenting with uveitis at a tertiary center in Switzerland over a ten year period.

## Methods

This is a single center retrospective study including data from 01.01.2012 til 30.06.2022 electronic health records (EHR) were searched automatically for patients under 18 years, presenting with uveitis. EHRs were manually reviewed for patients with positive informed consent. Data extracted included age at first presentation, sex, suspected infectious or non-infectious origin, primarily involved segment at first presentation, and laterality. Ocular function and morphology included: Habitual visual acuity (VA, Snellen, decimal), intraocular pressure (IOP, either with rebound tonometry or applanation depending on age and compliance), complications such as macular edema, optic disc swelling, epiretinal membrane, cataract, synechiae, band keratopathy, and glaucoma. Slit lamp exam was used for anterior segment examination, fundus exam was either done at the slit lamp and/or with indirect ophthalmoscopy. Optical coherence tomography was used with a low threshold to image the macula and the optic disc if a pathology was suspected. Wide field fundus photography and/or angiography was used in selected cases only. Extraocular and laboratory information was gained in collaboration with paediatric rheumathologists, paediatric infectiologists and other paediatric specialists and included: Associated systemic disease, antinuclear antibody (ANA) status, and human leukocyte antigen (HLA) B27 status. Diagnosis of ocular toxoplasmosis was based on typical clinical findings judged by an uveitis specialist; patients needed to have a positive serology for definite diagnosis. Diagnosis of herpetic uveitis was only based on clinical findings judged by an uveitis specialist. Documented symptoms reported by the patients were recorded. Patients were classified according to the SUN classification [3]. In patients were no infectious disease or associated systemic disease was found, the term “uveitis of unknown cause” was used and patients were classified as non-infectious uveitis. Statistics was performed using IBM SPSS Statistics. Frequencies and descriptive statistics were applied. Depending on the distribution, mean and standard deviation or median and interquartile range were computed. Non-parametric tests were used to compare samples. Significance level was set at *p* < 0.05. Local ethics committee approved this study (Institutional review board of Swiss Ethics/BASEC No. 2023 − 00439). The Tenets of the Declaration of Helsinki were followed.

## Results

**Fig. 1:**
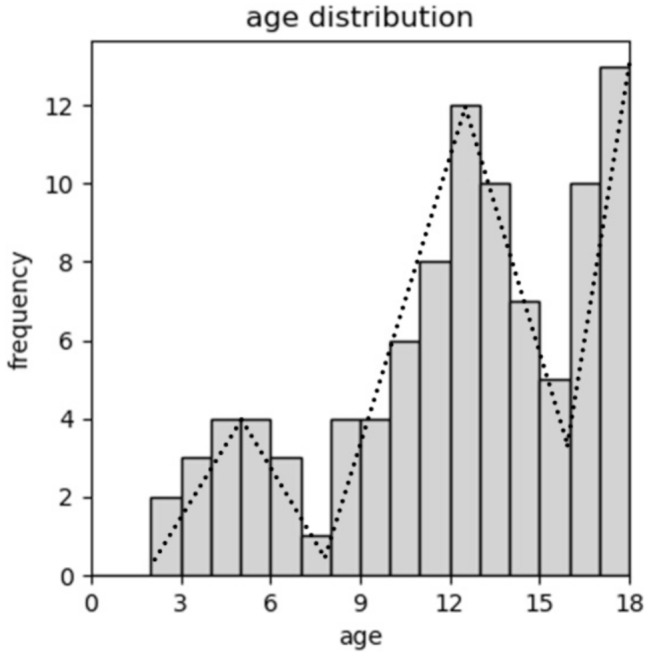
Age distribution at first presentation. There is a three peak distribution, with a first peak in preschool, a second in early teens, and a third in late adolescence

**Fig. 2:**
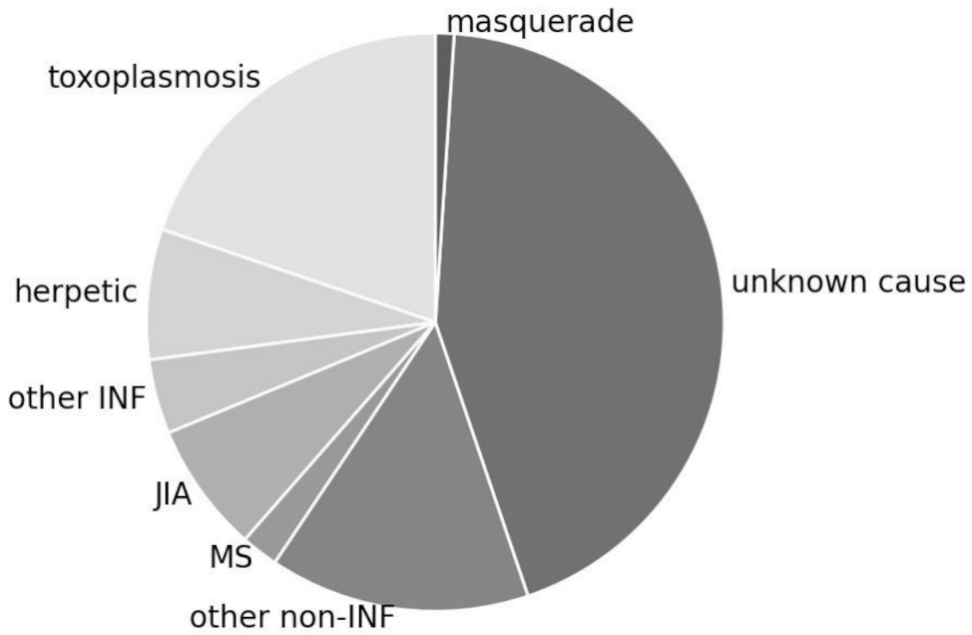
Piechart of uveitis etiologies or associated diseases. Infectious causes made up nearly one third, with toxoplasmosis being the most frequent etiology, followed by herpetic diseases. Other infectious causes were tick-born encephalitis, chlamydia urethritis, and gastrointestinal salmonella. 69% were found to be non-infectious uveitis, with 44% of unknown cause (whereof 11% were ANA positive and 5% were HLA B27 positive), 8% were JIA associated uveitis, 2% were later diagnosed with multiple sclerosis. Other associated diseases were Susac syndrome, TINU, SLE, Crohn’s disease, ankylosing spondylitis, familial Mediterranean fever. One patients was had a masquerade syndrome with ALL. INF, infectious; *ANA*, *antinuclear antibodies; HLA*, *human leukocyte antigen; JIA*, *juvenile idiopathic arthritis; MS*, *multiple sclerosis; TINU*, *tubulointerstitial nephritis uveitis syndrome; SLE*, *systemic lupus erythematodes; ANCA*, *anti-neutrophil cytoplasmic antibodies; ALL*, *acute lymphoblastic leukemia*

Out of 133 identified patients with paediatric uveitis between January 2012 and June 2022, 96 patients (56/93 with initial bilateral diseases, total of 149 affected eyes) could be included in the study. Median age at first presentation at our clinic was 12.0 years (range 2.0 to 17.6 years). There is a three-peaked age distribution with a first peak in preschool, a second peak in early teens, and a third in late teens as illustrated in Fig. 1. No female / male difference was found with 51% compared to 49%, respectively. Non-infectious etiology was apparent in more than double compared to infectious causes with 68% compared to 32%, respectively. Anterior uveitis was most common documented (50%), followed by posterior (28%), intermediate (20%), and panuveitis (2%). The overall most common etiology was toxoplasmosis (20%), followed by JIA (8%) and suspected herpetic infection (8%). Two female patients, both presenting with intermediate uveitis at age 17 years, were later (2 months and 2 years) diagnosed with multiple sclerosis (MS). One female patient with papillitis, 7 years of age, was diagnosed with tick-born encephalitis (TBE), confirmed by lumbar puncture. This child was not vaccinated. She presented complete recovery from the TBE. Another female’s anterior uveitis, presenting at 5 years of age, was preceded by salmonella diarrhea. It was considered to be a reactive uveitis and treated with topical steroids only, as the diarrhea has spontaneously ceased and there was no sign of invasive disease. Other associations were Susac syndrome (female, 17 years old), TINU syndrome (male, 13 years old), systemic lupus erythematodes (female, 15 years old), Crohn’s disease (male, 11 years old), ankylosing spondylarthritis (female, 13 years old), and familial Mediterranean fever (male, 12 years old) as summarized in Fig. 2.

One patient at age 13 years had a masquerade syndrome secondary to preexisting, still active acute lymphoblastic leukemia (ALL). No associated infectious cause or systemic disease was found in 44% of the cases, thereof 11% had elevated ANA and 5% tested positive HLA B27 (to note that HLA B27 tests were only documented in 60% of the patients). ANA patterns were analysed in eleven patients. In this sub cohort, no association between ANA pattern and systemic disease was found.

The distribution of associated diseases differed between the affected segments: Anterior uveitis was mostly associated with non-infectious causes (80%) whereas intermediate uveitis was always associated with non-infectious causes. Posterior uveitis was mostly of infectious cause with 81%. In contrast, panuveitis was always of non-infectious cause (see Table 1). Further, bilateral disease was strongly associated with non-infectious uveitis (93% of the cases), whereas unilateral disease was more likely to be of an infectious cause (70% of the cases), with an odds ratio of 31 (95% confidence interval 9–106) (see Table 2).

**Table 1:** Distribution of infectious and non-infectious uveitis according to the primarily involved segment

Segment	Overall patients	Cause		Most frequent etiology
		Non-infectious	Infectious	
anterior	46 (50%)	37	9	Herpetic (7) JIA (6) Other systemic autoimmune disease (7) - Systemic lupus erythematodes - Ankylosing spondylitis - Crohn’s disease - TINU Other infectios disease (2) - Chlamydia urethritis - Gastrointestinal salmonella Masquerade syndrome (1) Unknown cause (23)
intermedia	19 (20%)	19	0	MS (2) JIA (1) Other systemic autoimmune disease (3) - Familial Mediterranean fever - Immune defect Unknown cause (13)
posterior	26 (28%)	5	21	Toxoplasmosis (19) Other infectious disease (2) - Tick-borne encephalitis Other non-infectious causes (2) - Susac syndrome - Unclassified encephalitis Unknown cause (3)
panuveitis	2 (2%)	2	0	Unknown cause (2)

JIA, juvenile idiopathic arthritis; TINU, tubulointerstitial nephritis uveitis syndrome; MS, multiple sclerosis

**Table 2:** Cross-tabulation of laterality versus infectious and non-infectious uveitis

Frequency and percentage	Infectious uveitis	Non-infectious uveitis
Bilateral uveitis (56/93; 60% of all patients)	4 (7%)	52 (93%)
Unilateral uveitis (37/93; 40% of all patients)	26 (70%)	11 (30%)

Odds ratio of 31 (95% confidence interval 9–106)

Mean VA at first presentation of affected eyes was 0.79 (range hand motion to 1.6) and mean IOP was 13.6mmHg (range 5.5 to 28.0). VA was significantly worse in cases with infectious uveitis compared to non-infectious uveitis (0.64 versus 0.83, *p* = 0.017, Mann-Whitney-U test). There was no significant difference in IOP.

Complications were documented in nearly one third of all affected eyes (see Table 3). This included optic disc swelling (18%), synechiae (10%), cataract (5%), band keratopathy (3%), epiretinal membrane (2%), macula edema (1%), retinal neovascularisations (6%) and vitreous hemorrhage (1%). IOP was above 21 mmHg in five eyes at first presentation (3%). No patient presented with clinically significant hypotony. The overall presence of complications did not differ between in non-infectious and infectious uveitis cases (28% versus 29%). Complications affecting the anterior segment were more prevalent in non-infectious uveitis. In contrast, complications affecting the posterior segment were more prevalent in infectious uveitis, but without clinical significance when adjusting for the distribution of affected segments in infectious and non-infectious uveitis.

**Table 3:** Complications at first presentation

Complications	eyes *n* (percentage)
Anterior segment	
- Band keratopathy - Posterior synechiae - Cataract - IOP > 21mmHg	4 (3%) 15 (10%) 7 (5%) 5 (3%)
Posterior segment	
- Optic disc swelling - Epiretinal membrane - Macular edema - Retinal neovascularisations - Vitreous haemorrhage	27 (18%) 3 (2%) 2 (1%) 9 (6%) 1 (1%)

IOP, intraocular pressure

Symptoms expressed by the patients were documented in 88%. This included blurred vision (26%), floaters (19%), photophobia (17%), and headache (7%). A redness of the eye was noted by the patients or guardians in 39%. There was no significant difference in incidence of complications between the patients with or without complaints.

## Discussion

We present the demographic data of a Swiss paediatric uveitis cohort. Similar to other European cohorts, anterior uveitis and bilateral disease was most prevalent [1, 9]. 70% were non-infectious uveitis, including 44% of unknown cause.

In the non-infectious uveitis cases, JIA was the most common associated systemic disease. Literature including European and North American studies found similar results [9, 10]. This might differ for other continents, as prevalence differs between ethnic groups and countries [10, 11]. The EHR used in our cohort does not systematically record ethnicity; we could therefore not further evaluate this factor. Positive ANA titer were found in 71% of JIA associated uveitis. Positive ANA titer were also found in 11% without JIA or any other systemic disease. This represents about the amount of prevalence of positive ANA in healthy children without uveitis or JIA or other autoimmune disease [12, 13]. The clinical relevance of a positive ANA titer could be discussed for the affected patients once JIA or other autoimmune disease has been excluded by a paediatric rheumatologist. None of the patients in our cohort with positive ANA but no JIA at first presentation did develop JIA during follow up; this may have been prevented by the introduction of systemic treatment in 6 out of the 10 patients. It might be valuable taking note of the ANA patterns, as there seems to be an association of ANA pattern and rheumatic or non-rheumatic systemic diagnosis [14, 15]. In our cohort no association between ANA pattern and prevalence of associated systemic disease could be found, but this result has to be viewed with caution, as only a small subcohort could be analysed.

Two adolescents in our cohort with intermediate uveitis were diagnosed with MS. A study involving 122 children with intermediate uveitis with an onset under 16 years of age found MS as the etiology in 2.4% [16]. Of all patients with MS, an estimate of 2 to 5% have an onset in childhood or adolescence [17]. Similar to adults, ocular involvement mostly occurs as optic neuritis, followed by oculomotor palsies [17, 18]. Uveitis is not considered as a clinical activity of MS; but prevalence of MS is higher in patients with uveitis (and vice versa) than in the general population and therefore long term monitoring is preferable [16, 17].

Only one of the patients in our cohort was diagnosed with a masquerade syndrome. The patient had anterior uveitis in the context of ALL at age 13 years. Masquerade syndrome are reported to make out up to 9% of paediatric uveitis cases, and include retinoblastoma, leukemia, Coats disease, and retinal degenerative disorders among others [1]. ALL is the most common malignancy in childhood with a median age at first diagnosis of 15 years [19]. Direct ocular leukemic infiltration (as suspected in our case) is rare; Ocular involvement due to low platelet count is more common [20, 21]. Retinoblastoma seeding into the vitreous can mimic intermediate uveitis. One study grouping vitreous seeding in three classes found a median age of patients presenting ‘dust’ to be 11 years (range 3–41 years), whereas patients with ‘spheres’ and ‘clouds’ were significantly older (median 15.5 years and 32 years respectively), all of which could potentially be mistaken for intraocular inflammation [22]. Coats disease is mostly diagnosed within the first two decades of life, with mean age at diagnosis of 10 years [23]. Advanced Coats disease with extensive exudations and retinal detachment may be difficult to differentiate from other pathologies including uveitis [23]. Retinal degenerative diseases can mimic posterior uveitis at any age, particularly as reports suggest that many genetic retinal diseases involve an immune dysfunction [24]. Masquerade syndromes should always be part of differential diagnosis, especially in cases non-responsiveness to uveitis treatment.

Nearly a third in our cohort were of infectious cause, with toxoplasmosis being most frequent, followed by herpetic etiology (of note that these cases were clinically diagnosed and not confirmed by intraocular tissue sampling). Toxoplasmosis was overall the most frequent uveitis etiology in our cohort, in accordance with the findings of previous reports [9]. Herpes is a further common infectious cause in paediatric uveitis [2]. Other infectious causes in our cohort were one male adolescent with chlamydia urethritis related uveitis, emphasizing the importance of a complete medical history, including sexual activity and screening for sexual transmitted diseases in adolescents. Regarding the patient with tick-borne encephalitis associated uveitis, it is worthwhile mentioning that the Swiss government recommends TBE vaccine for anyone living in a tick risk region [25]. However the TBE vaccine is not part of the national vaccination plan and the costs are not covered by the compulsory health insurance. Regarding the management of uveitis in the setting of gastrointestinal salmonella infections, the indication of systemic antimicrobial treatment should best be discussed with the paediatrician, as any antibiotic might prolong excretion and is generally only indicated in invasive disease [26].

Presentations as uni - or bilateral disease as well as the involved segment helps differentiating between infectious and non-infectious uveitis. In our cohort, bilateral presentation was a high predictive factor for a non-infectious etiology and unilateral presentation was more likely to be of infectious etiology. Unilateral anterior uveitis was likely to be of herpetic origin, and unilateral posterior uveitis was always caused by toxoplasmosis in this cohort. Prevalence of infectious causes in paediatric uveitis is generally high, and should therefore always be taken into consideration, especially in unilateral cases, to adapt workup and treatment accordingly [2]. Our cohort does not present a difference for complications at baseline between infectious and non-infectious uveitis. Higher powered studies would be needed to investigate this in more detail. Unfortunately, prevalence of complications at first presentation is high in paediatric uveitis [9] which is confirmed by our cohort. This is thought to be due to the delays until uveitis is diagnosed due to frequent absence of complaints, preverbal age, difficulty in examining children as well as disease severity [4, 27]. Most of this sample’s patients did have complaints at first presentation. There was no relationship of incidence of complications at first presentation and report of complaints. It is not possible to determine the true onset of uveitis from the sample’s data; it may be that complaints developed with a certain delay.

## Conclusion

In this Swiss cohort, distribution of presentations and etiologies is similar to other European cohorts. Knowledge of demography is important for specialists to target workup, guide clinical diagnosis and timely induction of treatment in children presenting with uveitis, as differential diagnoses can be vast in this rare disease.

## Data Availability

The datasets analysed during the current study are available from the corresponding author on reasonable request. Any request to share the dataset will need to be reviewed with the local ethics committee.

## Abbreviations

VA: Visual acuity
JIA: Juvenile idiopathic arthritis
SUN: Standardization of uveitis nomenclature
TINU: Tubulointerstitial nephritis uveitis
EHR: Electronic health records
IOP: Intraocular pressure
ANA: Antinuclear antibody
HLA: Human leukocyte antigen
BASEC: Business Administration System for Ethics Committees
MS: Multiple sclerosis
TBE: Tick-born encephalitis
ALL: Acute lymphoblastic leukemia
